# Biodegradable Polylactic Acid and Its Composites: Characteristics, Processing, and Sustainable Applications in Sports

**DOI:** 10.3390/polym15143096

**Published:** 2023-07-19

**Authors:** Yueting Wu, Xing Gao, Jie Wu, Tongxi Zhou, Tat Thang Nguyen, Yutong Wang

**Affiliations:** 1 Graduate School, College of Sports and Human Sciences, Post-Doctoral Mobile Research Station, Harbin Sport University, Harbin 150008, China; wuyueting@hrbipe.edu.cn (Y.W.); wujie@hrbipe.edu.cn (J.W.); zhoutongxi@hrbipe.edu.cn (T.Z.); wangyutong@hrbipe.edu.cn (Y.W.); 2 College of Wood Industry and Interior Design, Vietnam National University of Forestry, Xuan Mai, Hanoi 13417, Vietnam; thangnt@vnuf.edu.vn

**Keywords:** PLA, biocomposites, biodegradation, sports equipment manufacturing

## Abstract

Polylactic acid (PLA) is a biodegradable polyester polymer that is produced from renewable resources, such as corn or other carbohydrate sources. However, its poor toughness limits its commercialization. PLA composites can meet the growing performance needs of various fields, but limited research has focused on their sustainable applications in sports. This paper reviews the latest research on PLA and its composites by describing the characteristics, production, degradation process, and the latest modification methods of PLA. Then, it discusses the inherent advantages of PLA composites and expounds on different biodegradable materials and their relationship with the properties of PLA composites. Finally, the importance and application prospects of PLA composites in the field of sports are emphasized. Although PLA composites mixed with natural biomass materials have not been mass produced, they are expected to be sustainable materials used in various industries because of their simple process, nontoxicity, biodegradability, and low cost.

## 1. Introduction

**Figure 1:**
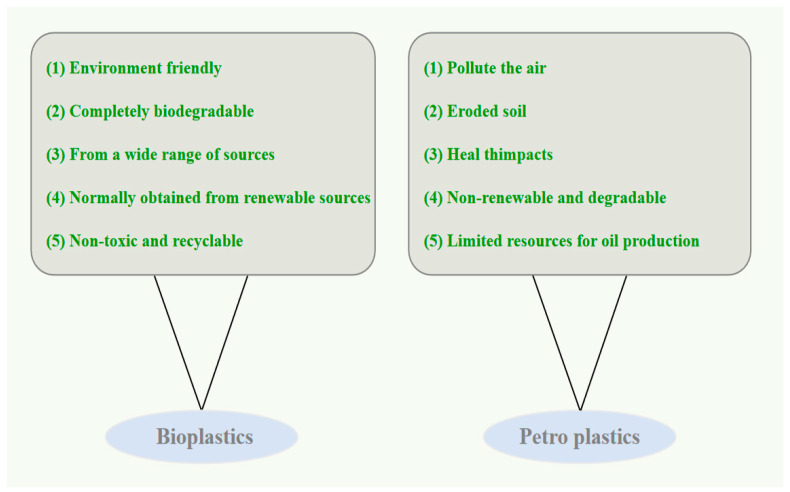
Properties of bioplastics and petro plastics (adapted from Refs. [6,7]).

The continuous advancement of science and technology has increased the global demand for natural resources, leading to frequent problems, such as material shortages and environmental pollution. Rapidly depleting oil reserves, greenhouse gas emissions, and the large-scale use of oil-based products have resulted in a lack of biodegradable products, prompting researchers to explore biodegradable, renewable, and recyclable materials. Polylactic acid (PLA) is a biodegradable bio-based aliphatic polyester that can be extracted from 100% renewable resources, such as corn, potatoes, and sugarcane [1]. Compared with traditional petroleum-based composite materials, PLA has a low density, low cost, good plasticity, and rigidity. PLA possesses excellent workability, making it an ideal choice for 3D printing sports equipment. 3D printing can be used to adjust the density and structure of a material according to specific requirements, allowing for personalized customization and innovative designs based on individual measurements and particular needs. This can achieve an ideal combination of lightweight and high strength to ensure that PLA sports equipment does not impose excessive burdens on athletes, enhances athletic performance, and protects different individuals. Although PLA possesses many characteristics suitable for the fabrication of sports equipment, more research is needed. Figure 1 compares the characteristics of bioplastics and petro plastics, showing that PLA occupies a crucial position in the biopolymer market and plays a vital role in various fields, such as in automotive, aerospace, construction, defense, food packaging, and sports equipment applications [2,3,4,5].

**Figure 2:**
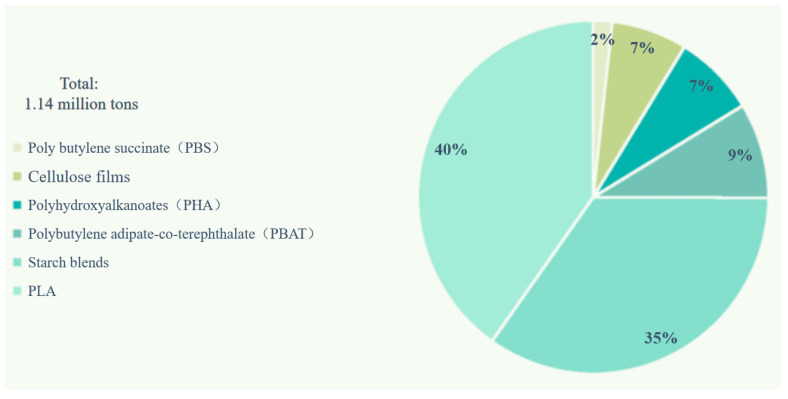
Global production capacity of PLA (adapted from Ref. [10]).

PLA is an extracted thermoplastic that is suitable for manufacturing composite materials using various methods, such as injection molding, extrusion molding, and compression molding [8]. Increases in the annual supply of PLA (Figure 2) and competitive petroleum costs are key factors driving researchers to develop new PLA-based biocomposites. PLA can biodegrade and bioaccumulate, which helps reduce production and waste disposal costs. PLA can also be treated by landfilling, incineration, or pyrolysis. More than 50% (2.8 kg CO_2_/kg PLA) of the released CO_2_ in the PLA life cycle is released during its conversion. By optimizing the conversion process of PLA, there is tremendous potential for PLA to become a low-carbon material [9].

This report discusses the latest developments in the research and development of PLA and its composites. It first outlines the basic structure, characteristics, production, degradation process, and latest modification methods of PLA, and it discusses the inherent advantages of selecting PLA composite materials and focuses on the relationship between different biodegradable materials and their performance in the final PLA composite materials. Then, it introduces the application prospects of PLA composite materials in the field of sports. Finally, it discusses the challenges faced by PLA composite materials and competing materials.

## 2. Overview of PLA

PLA is an entirely biodegradable polymer hailed as one of the most promising bio-based polymers because of its biocompatibility, biodegradability, high mechanical strength, nontoxicity, nonirritation, and processability. PLA can be synthesized by low-energy processes, and it is independent of petroleum resources. Microorganisms can decompose waste PLA into H_2_O and CO_2_. After photosynthesis, CO_2_ and water are converted back into substances such as starch, which can be used as raw materials to resynthesize PLA, thereby realizing a carbon cycle process [11] that does not pollute the environment.

### 2.1. PLA Structure

**Figure 3:**
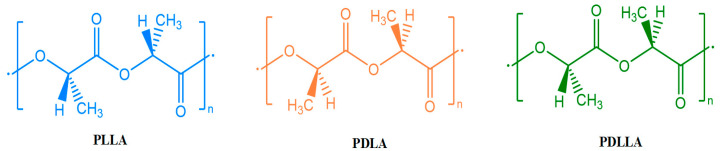
Three-dimensional configuration of PLA (adapted from Ref. [13]).

Lactic acid molecules contain a chiral asymmetric α-carbon atom and exhibit optical activity that can be divided into two configurations: left-handed (L) and right-handed (D). The dehydration of two lactic acid molecules forms three optical isomers of lactide: L-lactide, D-lactide, and meso-lactide. L-lactide is cheaper because it is naturally occurring. The content of D-lactic acid changes the crystallization behavior of PLA, including different crystallization rates, multiple crystal types, different scales, and layer thicknesses. The crystal morphology is closely related to the mechanical properties of the polymer: the larger the PLA crystal, the more defects in the interior and on the surface of the crystal, and the poorer the mechanical properties of the resulting material [12]. Like L-lactide, meso-lactide is a cyclic diester with two chiral carbon atoms that are not optically active. PLA synthesized via lactide ring-opening polymerization (ROP) has three different stereo configurations: left-handed polylactic acid (PLLA), right-handed polylactic acid (PDLA), and racemic polylactic acid (PDLLA) [13]. Figure 3 shows the three stereo configurations of PLA. The properties and applications of these PLA stereo configurations depend on the molecular weight, molecular weight distribution, crystal structure, and melt rheological behavior.

### 2.2. Properties of PLA

PLA is a member of the family of aliphatic polyesters and has the essential characteristics of universal polymer materials. PLA has a tensile strength similar to that of polyethylene terephthalate (PET), approximately 54 MPa, while its tensile modulus is 3.4 GPa, which is slightly higher than that of PET [14,15]. The mechanical properties of PLA are greatly affected by its molecular weight (*M*_w_). When the molecular weight doubles from 50 kDa to 100 kDa, the PLA’s tensile strength and elastic modulus also double [16]. The mechanical properties of PLA depend on its semicrystalline structure, amorphous structure, and crystallinity. Semicrystalline PLA shows greater mechanical properties than amorphous PLA. Upon increasing the PLA crystallinity and decreasing the molecular chain mobility, the elongation at the break of the material decreases, while the tensile strength and modulus increase. Table 1 shows the properties of PLA and its different stereo configurations. In addition, the stereochemical structure of lactic acid-based polymers can be controlled by copolymerizing L-lactide, D-lactide, D, L-lactide, and meso-lactide to slow the crystallization rate, which significantly impacts the mechanical properties [17,18].

The environmental degradation process of PLA occurs in two steps: hydrolysis and microbial degradation. PLA first undergoes the hydrolytic cleavage of ester bonds, degrading into PLA oligomers (OLAs) [19]. The hydrolysis of PLA can be catalyzed by acid or alkali and is also affected by temperature and humidity [20,21]. As hydrolysis proceeds, the number of –COOH groups in the system gradually increases, which plays a catalytic role in the cleavage of PLA ester bonds [22]. This makes the degradation of PLA a self-catalytic process. When the molecular weight of PLA decreases to below 10,000 g/mol, microorganisms can participate in the degradation process of PLA and eventually degrade it into H_2_O and CO_2_.

**Table 1:** Properties of PLA and PLA stereo configurations.

Polymer	Density (g/cm^3^)	Glass Transition Temperature (°C)	Melting Point (°C)	Molecular Weight (g/mol)	Tensile Strength (MPa)	Solubility	Refs.
PLA	1.25	54–56	120–170	66,000	21–60	Trimethylsilyl	[23,24,25]
PLLA	1.290	55–80	173–178	<350,000	15.5–150	Chloroform, furan, dioxane, and dioxole	[25,26,27,28,29]
PDLA	1.248	40–50	120–150	21,000–67,000	15.5–150	PLLA solvents, plus acetone	[26,27,28,30,31]
PDLLA	1.25	43–53	230–240	<350,000	27.6–50	Tetrahydrofuran, ethyl acetate, dimethyl sulfoxide, and dimethyl formamide	[25,26,27,28,29]

### 2.3. PLA Production

**Figure 4:**
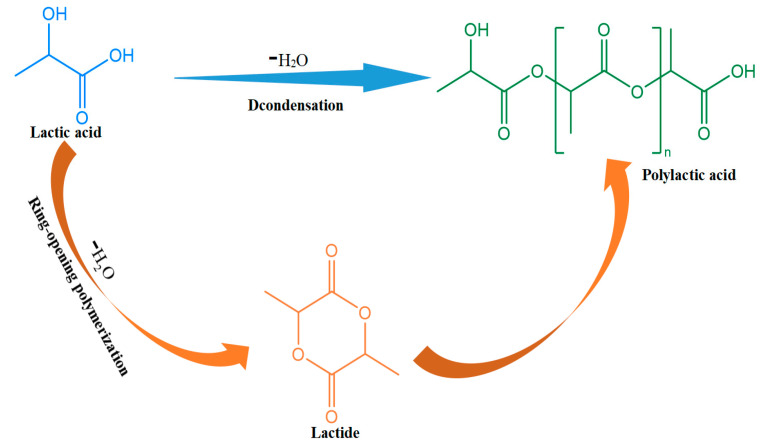
PLA synthetic pathways (adapted from Ref. [32]).

Figure 4 shows the synthetic route of PLA. Researchers extract starch from renewable natural resources, such as corn and potatoes, and ferment it to produce PLA. Traditional lactic acid fermentation uses starchy raw materials, and some countries have developed the use of agricultural and sideline products as raw materials for this process. The two main methods for synthesizing PLA are direct condensation and lactide ROP [32].

#### 2.3.1. Direct Polycondensation

In the late 1980s, the advancement of direct condensation technology significantly increased the global production of PLA and greatly reduced its costs. Direct condensation involves preparing PLA by dehydrating and condensing lactic acid molecules. The disadvantage of this method is that the reaction system is in a dynamic equilibrium between condensation and depolymerization, and the high viscosity of the system makes it difficult to remove the water by-product. The unremoved water causes the depolymerization reaction to proceed, even under vacuum conditions, making it difficult to extract water and increasing the molecular weight of the PLA. Under a high temperature (>200 °C), the PLA will undergo depolymerization, discoloration, and racemization accompanied by a series of side reactions, such as ester exchange, which may form differently sized cyclic products. This results in reduced product properties and poor mechanical properties, which limit their industrial applications. However, the use of direct condensation to produce PLA is a short and inexpensive method. Chen et al. used a combination of direct condensation and melt polymerization using tetra butyl titanate as a catalyst. They used different vacuum periods, esterification, and condensation reactions. The results showed that this method reduced the system’s viscosity, thus helping to remove water and increase the molecular weight [33].

#### 2.3.2. Ring-Opening Polymerization (ROP)

In the early 1990s, Cargill Inc. applied for a patent for a solvent-free process and new distillation technology based on ROP to convert lactic acid into high-molecular-weight polymers. This made PLA the second-highest volume bioplastic after starch-based materials. By utilizing specific microbial strains, natural agricultural materials can undergo fermentation to produce lactic acid (LA), which is a precursor for PLA [34,35]. The mature ROP process can make high-molecular-weight and chemically controllable PLA samples with good mechanical properties by controlling lactide’s purity and reaction conditions. This is currently the most common method for the industrial production of high-molecular-weight PLA. The technical difficulties of ROP production lie in the synthesis and purification of lactide. Lactide ROP, first, generates oligomers via the dehydration–condensation of lactic acid, and then oligomers are cracked into lactide using initiators, and the lactide, finally, undergoes ROP to generate PLA. Only high-purity lactide can be used to synthesize high-molecular-weight PLA with the desirable physical properties. Depending on the initiator used, lactide ROP can be divided into anionic, cationic, or coordination ROP. Among them, cationic ROP uses a smaller amount of catalyst, while anionic ROP has high reactivity and a fast speed [36].

### 2.4. Modified PLA

According to Refs. [37,38,39], the low flexibility, elongation, impact resistance, and heat distortion temperature of PLA results in problems such as low crystallinity, long injection molding cycle, high moisture sensitivity, and low hydrolysis resistance. Researchers have used different modification techniques to improve the performance of PLA, such as copolymers and blending with nanocomposites or other polymers.

#### 2.4.1. Copolymers

PLA is a thermoplastic polymer whose processing temperature is generally between 170 and 230 °C. In recent years, researchers have produced self-reinforcing PLA through techniques such as melt extrusion, stretching, and injection molding without the need for additives, which retain the biocompatibility and biodegradability of PLA. This method can also solve the trade-off between the toughness and strength and compatibility of blends. In addition, Cao et al. [40] designed a new modification process. After isothermal crystallization, blow molding was carried out below the melting point of crystalline PLA. A crystal network was formed through stretching and blow-molding to prepare a self-reinforcing PLA film. The elongation at the break of this film increased by approximately 67.50% and 104.83% in the transverse and longitudinal directions, respectively, and the tensile strength increased by approximately 45.4 MPa and 78.0 MPa in the transverse and longitudinal directions. This overcame the trade-off between the toughness and strength.

#### 2.4.2. Blending with Nanocomposites

Chrissafis et al. [41] added 2.5% oxidized multiwalled carbon nanotubes into PLA and found that the thermal stability of the modified PLA material was greater than that of pure PLA, and the thermal conductivity increased by about 60%. The hexagonal mesh structure and stable chemical bonds of oxidized multiwalled carbon nanotubes made them highly durable, with a decomposition temperature above 1000 °C. Since oxidized multiwalled carbon nanotubes disperse the heat absorbed by PLA, the modified PLA’s thermal conductivity and thermal stability were enhanced. In addition, oxidized multiwalled carbon nanotubes acted as heterogeneous nucleating agents in the PLA matrix. The growth of PLA crystals around the oxidized multiwalled carbon nanotubes shortened the induction process of PLA nucleation, accelerated the PLA crystallization, and reduced the spherulite size. Seligra et al. [42] grafted modified carbon nanotubes onto PLA, which significantly increased the conductivity of the modified PLA material to 4000 s/m. The added carbon nanotubes formed an electron-conducting network that lowered the percolation threshold, thereby transforming PLA into a conductive polymer. A small amount of carbon nanotubes was sufficient to increase the conductivity without affecting the material’s mechanical properties.

#### 2.4.3. Blending with Other Polymers

Researchers can improve the mechanical properties of polymers by changing the structure and composition of copolymers. Adjusting the ratio of lactic acid and other monomers in the copolymer system can produce copolymers with the desired mechanical strength to improve the mechanical properties of PLA. By utilizing the hydroxyl and carboxyl groups on the lactic acid segment, different monomers, such as caprolactone (CL), ethylene oxide (EO), ethylene glycol (EG), and trimethylene carbonate (TMC), can be used to synthesize PLA copolymers with improved mechanical properties, especially toughness. Li et al. [43] prepared alternating and random polyurethane copolymers using PLA and polyethylene glycol (PEG). The alternating polyurethane copolymer had a more controllable structure than the random polyurethane copolymer and, therefore, showed higher crystallinity and mechanical properties. Huang et al. [44] developed an electrochemically controlled switchable copolymer system and used it to quickly synthesize multisegment copolymers of PLA and polycarbonate propylene (PPC) without adding external oxidants or reducing agents. In this way, they exploited the complementary advantages of PPC (toughness) and PLA (mechanical strength).

### 2.5. PLA Degradation

PLA is a biopolymer that can also undergo biodegradation under certain conditions without producing environmental pollution [45,46]. Polymer degradation can be divided into heterogeneous and homogeneous degradation, also known as surface and intramolecular polymer degradation, which can occur through three different chemical reactions: (a) main-chain cleavage, (b) side-chain cleavage, and (c) cross-link cleavage. PLA degradation mainly occurs through ester bond cleavage, which splits long polymer chains into shorter oligomers, dimers, or even monomers. Specifically, the ester bonds of PLA are cleaved via chemical hydrolysis, and under the action of salicylic acid, they are split into carboxylic acids and alcohols. These shorter units are small enough to pass through the cell walls of microorganisms, where they serve as substrates for their biochemical processes and are degraded by microbial enzymes. PLA can be composted to produce CO_2_ and H_2_O, requiring temperatures near the *T*_g_ (60 °C) of the polymer and a high relative humidity [47]. The CO_2_ emissions are offset by the initial absorption during PLA production. Under such conditions, the degradation time can be as short as 30 days.

Piedmont and Gironi [48] studied the hydrolytic degradation kinetics of PLA at concentrations of 5–50 wt% between temperatures of 140 °C and 180 °C. The results showed that the reaction kinetics did not depend on the concentration of PLA, and the collected data indicated two different reaction mechanisms. The first mechanism was related to a biphasic reaction (*E*_a_ = 53.2 kJ mol^−1^), and the second mechanism was associated with a self-catalytic effect of increasing carboxylic acid groups during the depolymerization process (*E*_a_ = 36.9 kJ mol^−1^). This effect was previously noted in PLA hydrolysis and lowered the solution pH. The group’s further work modeled the hydrolysis of PLA at higher temperatures (170–200 °C). The kinetic model described the batch erosion of PLA and subsequent hydrolysis of oligomers, and the model accurately predicted the conversion and concentration of oligomers. Under these conditions, PLA could be completely transformed within 90 min.

## 3. PLA Composite Materials

**Figure 5:**
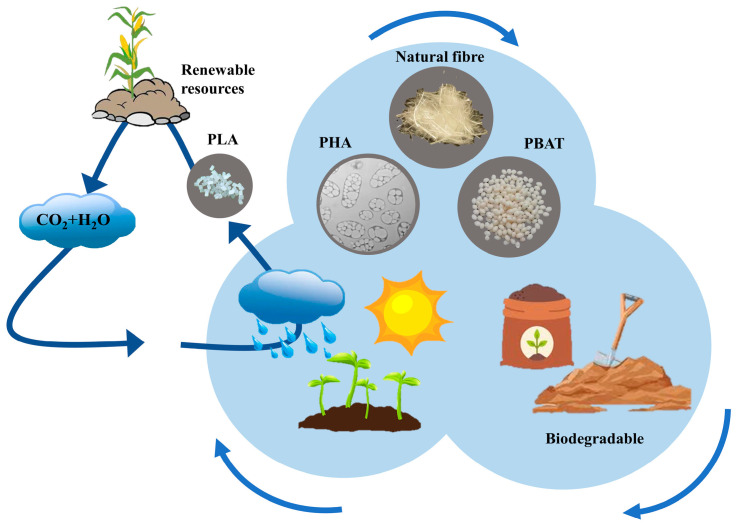
PLA composite degradation process (adapted from Refs. [46,47,57]).

Although PLA has excellent mechanical properties, renewability, biodegradability, and low costs [49], Figure 5 shows PLA composite degradation process, it is also brittle and has low heat resistance [50]. Researchers have explored various reinforcement materials to develop PLA composite materials to overcome these drawbacks [51], such as cellulose, lignin, silk, PBAT, and PHA. Table 2 compares the mechanical properties of different PLA composite materials.

**Table 2:** Comparison of the mechanical properties of different PLA composites.

Reinforcement	Addition of Fiber (wt.%)	Best Combination (wt.%)	Tensile Strength (MPa)	Tensile Modulus (GPa)	Ref.
Cellulose	30	-	62.3	4.1	[52]
Wood flour	20–40	30	63.3	5.3	[53]
Silk	1–7	5	62.08	2.54	[54]
PBAT	20	-	66.1	1.078	[55]
PHA	20	-	25.4	1.2	[56]

### 3.1. Natural Fibers

Natural fibers can be divided into plant and animal fibers according to their sources [58]. Generally, combining natural fibers with PLA significantly improves the tensile strength, flexural strength, elastic modulus, heat distortion temperature, and other properties of PLA composites. This also enhances their impact resistance and dimensional stability [59,60] while reducing costs. Therefore, natural fibers are an ideal choice for preparing PLA composite materials.

#### 3.1.1. Cellulose Nanocrystals

Cellulose nanocrystals (CNCs) are rod-shaped nanoparticles extracted from cellulose through acid hydrolysis. A wide range of sources, including bleached wood pulp, cotton, and hemp fibers, can be used to produce CNCs [61,62]. Because of their high specific surface area, high reactivity, high strength, and low density, CNCs are an attractive reinforcement material.

Since Favier et al. [63] first attempted to use cellulose whiskers to reinforce polymers in 1995, nano cellulose products have been commercialized, which has prompted researchers to develop PLA/CNCs composite materials. Most studies have shown that CNCs can be well dispersed in PLA and act as a heterogeneous nucleating agent that affects the crystallization of PLA [64]. During isothermal or nonisothermal bulk crystallization, the presence of CNCs reduces the activation energy of PLA crystallization and increases the crystallization rate of PLA. Kamal et al. [65] prepared CNCs/PLA composites by melt blending and found that CNCs acted as a heterogeneous nucleating agent that promoted the formation of PLA crystals, increased the crystallization rate, and improved the crystallinity. Karkhanis et al. [66] used CNCs to prepare packaging film with PLA composites. Compared with a PLA film, the water vapor permeability of the composite film decreased by 40%. The oxygen permeability decreased by 75%, thus significantly improving the barrier properties of the thin film. The presence of numerous hydroxyl groups on the surface of CNCs controlled the degradation performance of the material and enhanced the hydrophilicity of PLA composites. Shuai et al. [67] introduced CNCs into a laser-sintered PLA scaffold and found that CNCs, as a heterogeneous nucleating agent, caused the ordered arrangement of PLLA chains by forming hydrogen bonds between the surface hydroxyl groups of CNCs and PLLA, thereby increasing the crystallization rate and crystallinity. In addition, since the mechanical strength of polymers is closely related to their crystallinity, the addition of 3 wt% CNCs to the PLA scaffold increased its compressive strength, compressive modulus, tensile strength, tensile modulus, and Vickers hardness by 191%, 351%, 34%, 83.5%, and 56%, respectively. Adding hydrophilic CNCs also improved the hydrophilicity and degradation performance of PLLA.

#### 3.1.2. Lignin

Lignin is the most abundant aromatic biomass in nature, accounting for 20–30% of the weight of wood [57,68]. Most natural lignin (approximately 98%) is currently unused as a value-added product and is discarded as industrial waste because its chemical structure in its raw form is fragile and lacks resistance to heat, chemicals, external loads, and other factors. When lignin is mixed with organic polymers, acetylation reactions reduce the strength of the hydrogen bonds in lignin molecules, thereby reducing the size of the structural domains when polymerized lignin is mixed with organic polymers [69]. Interactions between the hydroxyl groups of lignin and the carboxyl groups of PLA underpin the production of PLA/lignin composite materials [70].

Spiridon et al. [71] obtained PLA/lignin biocomposites by melt blending, and a study of the impact of their physicochemical parameters showed that adding different concentrations of lignin increased the Young’s modulus and tensile strength of the material. PLA/lignin biocomposites showed excellent mechanical resistance, remained stable during a 30-day degradation process, and maintained their dimensional stability in fluid environments. In addition, lignin did not cause cytotoxicity, demonstrating that PLA/lignin biocomposites have good biocompatibility. Tanase-Opedal et al. [72] studied the 3D printing of PLA/lignin biocomposites. Because of the antioxidant activity of lignin, PLA/lignin biocomposites showed incredibly high antioxidant activity, good extrudability, and excellent flowability, making them a promising renewable substitute for traditional 3D printing materials.

#### 3.1.3. Silk Fiber

Silk fiber is a natural animal protein fiber with a higher crystallinity, toughness, and tensile strength than plant fibers [73]. In addition to having good mechanical properties and biocompatibility, silk fiber is also easier to process. However, its softness may limit its applications in fields that require high hardness and rigidity. Therefore, it is necessary to optimize the properties of silk fiber for specific applications, including by mixing it with other materials such as PLA to produce tough and rigid materials [74] with improved mechanical properties. Silk/PLA composites may also show greater biocompatibility, making them suitable for various sports medicine and bioengineering applications.

Zhao et al. [75] prepared silk/PLA biocomposites by melt blending and found that adding silk fiber improved the dimensional stability. The presence of silk fiber also enhanced the enzymatic degradation of the PLA matrix, thereby controlling its susceptibility to hydrolysis. Cheung et al. [76] studied the mechanical properties and thermal behavior of silk/PLA biocomposites and found that their tensile performance was superior to that of pure PLA. Therefore, adding silk fiber improved the thermal and physical properties of the composite, making it suitable for use in medical scaffolds.

### 3.2. PHA

As a new bio-based polymer material, PHA has diverse structures, various sources, and biodegradability, biocompatibility, optical activity, piezoelectricity, and gas barrier properties. They can be naturally biodegraded into CO_2_ and H_2_O and are nontoxic to the soil and air [77,78]. Currently, over 150 different PHA monomers have been discovered and produced by other bacteria and growth conditions of which PHB, PHBV, PHBHHx, and P34HB are the four main types. The discovery of these different PHA monomers has dramatically increased the development of PHA into commercial plastic products [79,80].

Zembouai et al. [81] studied PHBV/PLA blends with different mass ratios and found that PHBV acted as a nucleating agent for PLA, thus improving the crystallization of PLA, and the tensile strength and elongation at the break of PHBV/PLA blends were higher than those of pure PHBV. ePHA is a PHA belonging to the polyhydroxy fatty acid family with the same chemical structure, biodegradability, and renewability. Takagi et al. [82] prepared PLA/PHA blends with different compositions by mixing PLA with PHA and functionalized ePHA containing 30% epoxy groups in the side chains. They found that the Charpy impact strength of the PLA/PHA and PLA/ePHA blends increased with the PHA or ePHA content and was higher than that of pure PLA. Functionalizing ePHA with epoxy side groups enhanced the compatibility of the mix, thereby increasing the tensile strength and Charpy impact strength of the PLA/ePHA mixture. The blending of PHA and PLA improved the properties of PHA and also guaranteed the degradability of the composite material.

### 3.3. PBAT

PBAT is a biodegradable material produced on large scales and widely used in packaging materials and biomedical fields. PBAT has good processability and can toughen and modify other polyesters [83], but commercially available PBAT/PLA blends often exhibit macroscale phase separation and show two glass transition temperatures (*T_g_*), indicating the poor compatibility of unmodified PBAT/PLA blends. In experimental studies, the preparation of PBAT/PLA blends usually involves melt blending. At high temperatures and sufficient time, ester exchange reactions occur between the two polyesters, thereby improving their compatibility [84]. By increasing the PBAT content within a specific range, the mechanical properties of PLA/PBAT composites, such as impact strength and elongation at break, can be improved [85].

Arruda et al. [86] prepared PLA/PBAT blends using an epoxy-functionalized chain extender and investigated the effect of 0.3% and 0.6% chain extenders on the mechanical properties, thermal properties, and microstructure of PLA/PBAT blends with ratios of 40/60 and 60/40. In the blend containing 40% PLA and no chain extender, the microstructure was significantly affected by the chain extender. PLA exhibited a fibrous dispersed phase, appearing elongated in the film stretching direction. In the mixture containing 60% PLA and no chain extender, PBAT displayed a large, belt-like structure in the middle of the film, with an overall skin-core design. The chain extender increased the crystallization temperature of PLA in both blends with different ratios and reduced the crystallinity of PBAT.

### 3.4. Methods for Manufacturing PLA-Based Composites

#### 3.4.1. Microcellular Injection Molding

Microcellular injection molding was first proposed in the 1980s by Nam et al. [87]. The formation of pores in microcellular foams proceeds via four main stages: construction of a polymer/supercritical fluid homogeneous system, bubble nucleation, bubble expansion, and cooling and solidification [88,89,90,91]. Microcellular foam injection molding can be used to produce microcellular foam products with micropores, with millions of pores per unit volume. Compared with nonfoamed substrates, microcellular foam materials exhibit at least a four-fold higher fracture toughness and impact resistance [92].

#### 3.4.2. Extrusion Molding

Extrusion molding can be divided into continuous and intermittent types based on the different pressures used during extrusion. Continuous extrusion applies pressure with the rotation of a screw to uniformly plasticize the material inside the barrel. The material undergoes mixing and heating through the action of the screw during the extrusion process, resulting in good material uniformity [93]. Intermittent extrusion applies pressure to the material through a plunger. While this provides a higher pressure than screw extruders, its ability to generate significant shear action is limited, and its operation is discontinuous, which limits its application range [94].

#### 3.4.3. Compression Molding

Compression molding is a standard processing method for PLA. During compression molding, PLA particles are placed in a heated mold, and pressure is applied to liquefy and flow the material at high temperatures [95]. As the material cools, it resolidifies and shapes the mold. The final product’s body, size, and performance can be controlled by adjusting the temperature, pressure, and holding time. Compared with other molding methods, compression molding has lower mold fabrication costs [96].

## 4. PLA Composites for Sports Applications

**Figure 6:**
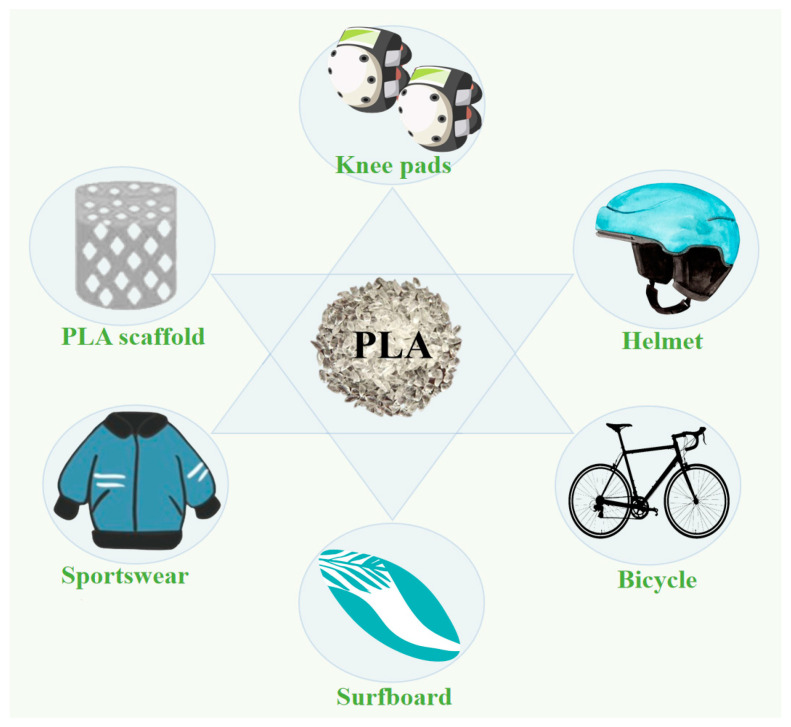
PLA applications in the sports industry.

The global production capacity of all biodegradable plastics, including PLA, is expected to increase rapidly to approximately 1.33 million in 2024 [6], with primary applications in the automotive industry, electronic components, and sports equipment. In the automotive industry, 3D printing has had a revolutionary impact by enabling the rapid fabrication of lighter and more complex structures. For instance, in 2014, Local Motors manufactured the first electric car using 3D printing. The automotive industry utilizes 3D printing during the improvement stage to explore various alternative solutions to promote ideal and efficient car design. 3D printing can also reduce material waste and consumption [97]. Because of the ability of 3D printing to create highly integrated three-dimensional multifunctional structures, many researchers have actively explored this emerging technology to fabricate geometrically complex and biocompatible devices and scaffolds. These include biosensors, electrically stimulated tissue-regenerating scaffolds and microelectrodes [98,99]. New technologies for producing high-molecular-weight PLA have expanded their applications in recent years. PLA is becoming a popular substitute for petroleum-based synthetic polymers (PETs, polystyrene (PS), polyethylene (PE), etc.) in various fields, particularly the sports industry [100,101], as shown in Figure 6.

### 4.1. Sportswear

PLA fiber is a biodegradable synthetic fiber that is refined and fermented from starch sugar in corn, beets, or wheat. It is a new type of polyester fiber in the textile industry. PLA fiber is 100% compostable and reduces the Earth’s carbon dioxide levels throughout its entire life cycle. The cross-section of PLA fiber is generally circular with a smooth surface. Its load–elongation curve is similar to that of wool, while its toughness is lower than that of cotton. PLA fiber has good core absorbency and fast moisture management. Therefore, by blending PLA fiber with cotton, the moisture transmission properties of cotton fabrics can be improved. Guruprasad et al. [102] developed a sports textile by combining cotton and PLA at a ratio of 65:35. Then, they tested the moisture management performance, moisture vapor transmission rate, and thermal performance of the cotton/PLA blended fabric. Experiments showed that the mixture of PLA fiber and cotton provided improved moisture management performance. The liquid transfer rate of cotton/PLA blended fabric was faster than that of 100% cotton fabric. The cotton/PLA composite fabric had a high unidirectional transmission capacity, spreading speed, and bottom absorption rate, giving it a higher OMMC value and allowing it to transfer sweat to the other side faster. The moisture vapor transmission rate of the cotton/PLA blended fabric was 14% higher than that of the 100% cotton fabric, which helped liquid moisture diffuse quicker, making it an ideal material for sportswear.

### 4.2. Helmets

Raykar et al. [103] used PLA plastic to manufacture a bicycle helmet through a combination of fused deposition modeling (FDM) and 3D printing. PLA plastic filaments were used and melted and deposited using layer-by-layer heat extrusion onto the building platform of the 3D model until the entire exterior of the helmet was covered in PLA plastic. After cleaning and trimming, a PLA bicycle sports helmet was produced. Experiments proved that the 3D printed PLA bicycle sports helmet had high safety, good breathability, and lighter weight, thus balancing the safety and comfort of the athlete.

### 4.3. Protective Sports Gear

Traditional protective sports gear has a structure consisting of a hard outer shell made of a thermoplastic material and an inner soft foam padding. Currently, there are new “soft shell” technologies for sports protectors based on the use of soft polymer foams typically made of polyurethane or polyacrylate with good cushioning properties. During the manufacturing process of sports protectors, soft polymer foams can be combined with PLA. Soft polymer foams are used as the internal cushioning material. In contrast, PLA can be used as the outer shell material to improve sports knee protectors’ lightweight, breathability, and comfort properties, thus achieving better protection results. Yang et al. [104] used tensile materials (PLA and thermoplastic polyurethane (TPU)). They tested them through 3D printing prototyping and compared the results with calculated predictions to evaluate the possibility of using tensile materials in sports protectors. The results showed that the tensile material had a high fracture toughness, high shear modulus, superior specific strength, compressive indentation resistance, strong energy dissipation, and a controllable strain penetration rate, making it suitable for protective sports gear to reduce the risk of injuries.

### 4.4. Surfboards

The source of power for a surfboard comes from the movement of waves, and the significant impact force generated by an impact wave can often break a surfboard, mainly when materials such as fiberglass are used in its production. In recent years, researchers have turned their attention to biodegradable materials. Soltani et al. [105] used the finite element method (FEM) and 3D printing to manufacture a surfboard with a uniform honeycomb core structure based on a PLA composite material. They then conducted three-point bending experiments and used accurate finite element tools to simulate surfboards with different core structures. The PLA composite material surfboard passed the three-point bending test, and the overall volume of the surfboard remained unchanged.

### 4.5. Sports Medicine Tools

Because of the biocompatibility and biodegradability of PLA when in contact with mammalian bodies, it has been widely used in the biomedical and pharmaceutical fields [106] to manufacture screws, pins, surgical sutures, stents, etc. [107,108]. The unique properties of PLA make it suitable for reinforcing rotator cuff repairs and can help heal tendon tissues in various body parts. PLA and its copolymers are often used in orthopedic surgery to manufacture artificial bones and joints, providing a temporary structure for tissue growth, which eventually decomposes. Koh et al. [109] used PLA-reinforced suture anchors to suture and repair tendons separated from the bone. The tensile strength of PLA is approximately 1200 N, and it can be manufactured to the required size. The experiment showed that adding a PLA scaffold to the bone bridge increased the fixation strength by 1.3 times. The use of PLA scaffolds showed significant advantages when used to fix the rotator cuff.

### 4.6. 3D Printed Sports Equipment

Compared with traditional printing materials, PLA produces almost no harmful gases and has a lower shrinkage rate, making it ideal for 3D printing sports equipment. Protective gear, such as mouthguards, helmets, and shin guards [110], can be 3D printed using PLA, providing athletes with customizable, comfortable, and lightweight equipment. Because of its biocompatibility, PLA is the preferred material for 3D printing protective gear, as it can be safely used in contact sports without causing harm to athletes. In addition to protective gear, PLA can be used to 3D print bicycle frames, kayak paddles, and skis [111]. PLA’s mechanical properties and biodegradability make it an attractive alternative to durable materials, such as plastics, metals, and other traditional materials for sports applications.

### 4.7. Limitations of PLA Composites in Sports Applications

Compared with traditional petroleum-based plastic sports equipment, the green disposal of idle sports equipment meets the requirements of sustainable development. Sports equipment made of PLA composites can be used safely and decomposes after being discarded, which can prevent environmental pollution. In addition, PLA has a lower density, allowing for the production of relatively lightweight sports equipment. Through 3D printing, PLA enables personalized customization, offering more possibilities for the innovative design of sports equipment. However, as a linear thermoplastic polyester, PLA’s strength may not meet the requirements of certain sporting equipment in specific environments. For example, because of PLA’s high brittleness and low elongation at break [25], sports equipment made from PLA composite materials are more susceptible to rupturing during contact sports. Additionally, prolonged exposure to sunlight can cause a decrease in the molecular weight of PLA composite materials [112], potentially impacting the mechanical performance of outdoor sports equipment.

## 5. Conclusions

PLA is a natural, renewable, and low-cost biodegradable material, but its inherently poor toughness limits its broader applications. By adding reinforcement materials to develop PLA composites, it can adapt to the increasing performance requirements of various fields. Compared with most inorganic and synthetic fibers, natural fibers have abundant sources, low prices, complete degradability, low energy consumption, and environmental friendliness. In the future, appropriate additives, modifications to polymerization conditions, and reinforcement techniques will be employed to enhance the strength of PLA and meet specific needs. At the same time, by developing low-cost reinforcement materials and optimizing formulations and processing methods, the manufacturing costs of PLA composites can be reduced. Their performance can be improved to meet various environmentally friendly applications, including sports equipment manufacturing. Currently, the application of PLA composites in the sports field is expanding. Compared with petroleum-based materials, the mechanical properties of PLA composites still need to be improved. However, as biodegradable alternatives to petroleum-based plastics, they still have tremendous potential.

## Data Availability

Not applicable.
